# Fisher–Shannon Complexity Analysis of High-Frequency Urban Wind Speed Time Series

**DOI:** 10.3390/e21010047

**Published:** 2019-01-10

**Authors:** Fabian Guignard, Dasaraden Mauree, Michele Lovallo, Mikhail Kanevski, Luciano Telesca

**Affiliations:** 1 IDYST, Faculty of Geosciences and Environment, University of Lausanne, CH-1015 Lausanne, Switzerland; 2 Solar Energy and Building Physics Laboratory, Ecole Polytechnique Fédérale de Lausanne, CH-1015 Lausanne, Switzerland; 3 Agenzia Regionale per la Protezione dell’ Ambiente di Basilicata, 85100 Potenza, Italy; 4 National Research Council (CNR), Institute of Methodologies for Environmental Analysis, C.da S.Loja, 85050 Tito (PZ), Italy

**Keywords:** high-frequency wind speed measurements, Fisher–Shannon complexity, time series, urban areas

## Abstract

One-hertz wind time series recorded at different levels (from 1.5–25.5 m) in an urban area are investigated by using the Fisher–Shannon (FS) analysis. FS analysis is a well-known method to gain insight into the complex behavior of nonlinear systems, by quantifying the order/disorder properties of time series. Our findings reveal that the FS complexity, defined as the product between the Fisher information measure and the Shannon entropy power, decreases with the height of the anemometer from the ground, suggesting a height-dependent variability in the order/disorder features of the high-frequency wind speed measured in urban layouts. Furthermore, the correlation between the FS complexity of wind speed and the daily variance of the ambient temperature shows a similar decrease with the height of the wind sensor. Such correlation is larger for the lower anemometers, indicating that ambient temperature is an important forcing of the wind speed variability in the vicinity of the ground.

## 1. Introduction

When designing urban areas, it is fundamental to consider multiple meteorological parameters. In particular, the buildings and the layout of the urban spaces will strongly impact the pedestrian comfort, building energy use [1,2], dispersion of air pollutants, and renewable energy potential in urban planning scenarios [3] due to the wind flow around the built-up spaces. Indeed, the wind flow around buildings is considerably different from the classical logarithmic wind profile [4] that can be observed in the surface layer in the boundary layer. Above a plane surface or in the absence of obstacles, the logarithmic profile provides a good approximation for the average wind speed. This is not the case in an urban boundary layer, where the complexity of the environment (high density of vertical surfaces, presence of various obstacles: trees, buildings, urban furniture) gives rise to additional turbulent structures [5] that cannot be simply parameterized. Therefore, there is a need to understand better the influence of the obstacles (buildings, trees, street equipment) on the airflow. This can only be achieved with high-quality and high-frequency wind data that are registered through experimental campaigns and field trips. Classical meteorological or climatic stations do not measure the wind speeds and direction with a sufficiently high vertical resolution and high frequency. The Basel Urban Boundary Layer Experiment (BUBBLE) [6] campaign in the early 2000s provided useful data for the development and generalization of new parameterization schemes. However, one urban configuration and such a short observation period are not enough to generalize formulations and to understand the underlying physical processes. For instance, to determine the momentum and heat fluxes, it is necessary to measure the vertical profiles of wind speed near buildings [7,8,9]. Additionally, urban configurations are often characterized by small-scale turbulence [10]. Only high-frequency wind speed data allow the identification of such turbulence.

The complexity of the wind speed in urban areas can be related to the nonlinear interactions that take place at any timescale between the average wind speed, vertical gradient, turbulent processes, shape, size and setting of buildings, etc. Hence, robust statistical methodologies are necessary to better characterize the time variability of wind speed in urban areas at different heights from the ground [11,12]. The presence of heterogeneous artificial or natural surfaces close to the ground significantly increases the complexity of the turbulent structure. As an example, the high density of vertical surfaces and the ground heating could lead to the development of thermal instabilities.

**Figure 1:**
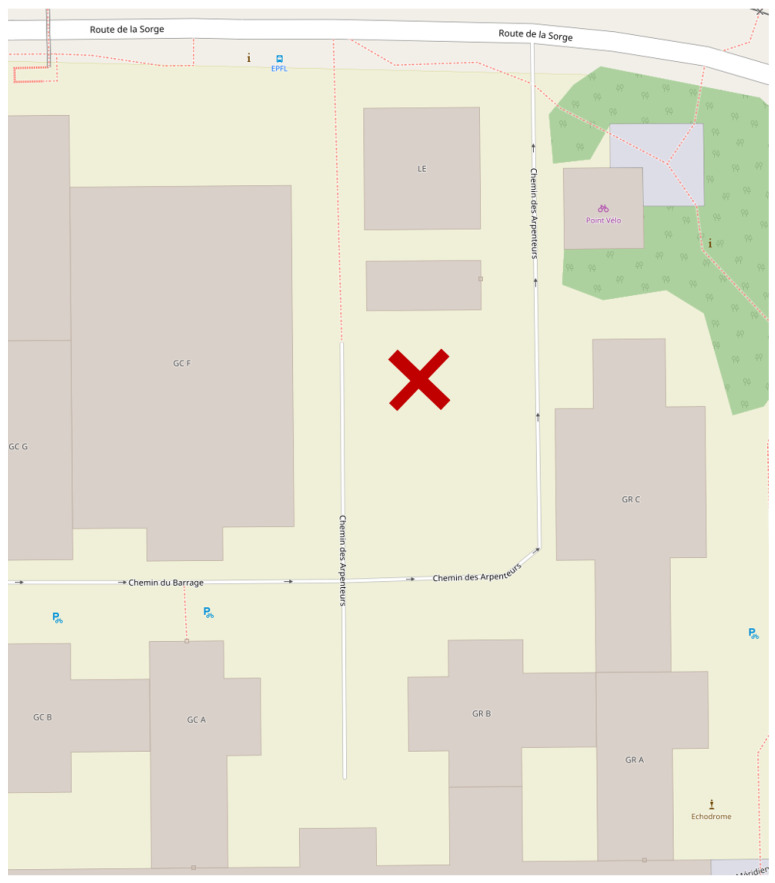
Location of the mast. This image is taken and modified from Open Street Map whose copyright notices can be found here: https://www.openstreetmap.org/copyright (CC-BY-SA-2.0).

In this study, we investigate the properties of order/disorder in the time variability of seven 1-Hz urban wind speed time series measured at different levels from the ground (from 1.5 m–25.5 m, with 4-m spacing between each anemometer), on a 27 m-high mast located on the campus of Ecole Polytechnique Fédérale de Lausanne (EPFL), Switzerland (motus.epfl.ch). The average height of the building layout around the mast is about 10 m [11,12]; see Figure 1. The experiment was performed with the aim to evaluate quantitatively how urban buildings could impact the wind. Building layouts generally cause local turbulence phenomena in the wind flow below the average height of the buildings. Thus, the main goal of the experiment was to discriminate between the turbulent dynamics of wind speed recorded by the anemometers installed below the average building height from the “free flow” dynamics of wind speed recorded by the anemometers placed above. To this aim, in the present research, the Fisher–Shannon method is used. This method jointly uses both Fisher information and Shannon entropy on time series. Fisher–Shannon analysis has some useful applications, e.g., it allows detecting non-stationarity [13] and leads to a measure of complexity [14]. The Fisher–Shannon method has been previously used on wind measurements [15,16].

The paper is organized as follows. First, a brief description of the experiment is presented. Then, the Fisher–Shannon method is explained. Next, the results are discussed, and the final remarks are summarized in the conclusions.

## 2. Description of the Experiment

**Figure 2:**
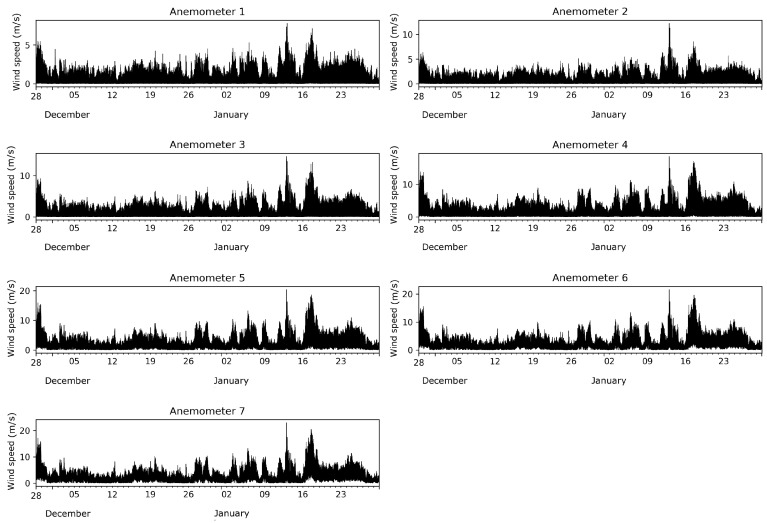
One-hertz wind speed time series for the seven anemometers.

**Figure 3:**
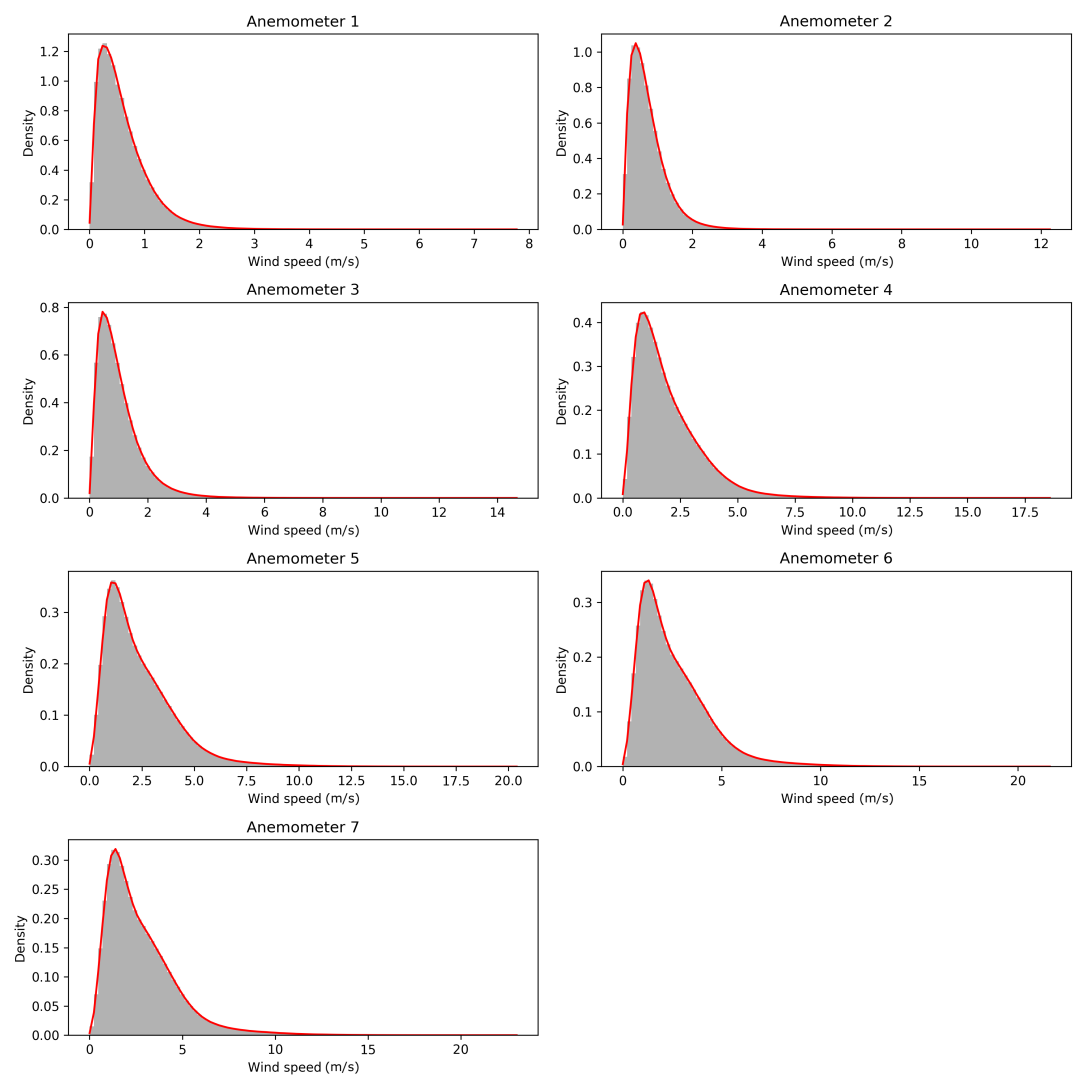
Histograms and kernel density estimations for the seven anemometers.

A meteorological mast of 27 m in height, where seven sonic anemometers (Gill Wind Master) are located, each 4 m, has been installed on the EPFL campus. The first anemometer is mounted at 1.5 m (corresponding to the average height of the center of an adult), while the last one is at 25.5 m above the ground. It can be noted that the highest sensor was placed far enough above the building layout height to be in the inertial layer [17]. The three velocity components, the sonic speed, and temperature were measured. The frequency of the data is 1 Hz [18]. In this study, the wind speed and the sonic temperature are analyzed for each level on the tower. The atmospheric pressure is measured at the site, with a Gill meteorological station (GMX300), also with a frequency of 1 Hz. The time series of the wind speed data, collected during a two-month period from 28 November 2016–29 January 2017, are shown in Figure 2. Figure 3 shows histograms along with kernel density estimations [19,20]. Summary statistics are given in Table 1.

## 3. The Fisher–Shannon Analysis

The Fisher–Shannon (FS) method is based on the analysis of two quantities, namely the Fisher Information Measure (FIM) and the Shannon Entropy Power (SEP).

Let *X* be a random variable and f(x) be its probability density function (pdf). The FIM of *X* is the real number I(X) defined as [21]:

(1) $$I\left(X\right)=\int_{-\infty }^{\infty }{\left(\frac{\partial }{\partial x}\log f\left(x\right)\right)}^{2}f\left(x\right)dx.$$

FIM quantifies the amount of organization in the data. The SEP of *X* is the real number, denoted N(X), defined as [22]:

(2) $$N\left(X\right)=\frac{1}{2\pi e}e^{2H(X)},$$

where H(X) is the differential entropy of *X* given by:

(3) $$H\left(X\right)=-\int_{-\infty }^{+\infty }f\left(x\right)\log f\left(x\right)dx.$$

SEP is a measure of disorder in data.

Note that FIM and SEP only depend on the pdf f(x), which requires being estimated. This estimation is performed by the kernel density estimator approach [23]: for a given time series $\left\{x_{i}\right\}$ of length *L*,

(4) $${\hat{f}}_{b,L}\left(x\right)=\frac{1}{bL}\sum_{i=1}^{L}K\left(\frac{x-x_{i}}{b}\right),$$

where *b* is the bandwidth and K(u) is the kernel function, which is assumed to be continuous, non-negative, symmetric around zero, and satisfying the following constraint:

(5) $$\int_{-\infty }^{+\infty }K\left(u\right)du=1.$$

The computation is carried out by combining the algorithms from [24,25], which use a Gaussian kernel with zero mean and unit variance:

(6) $${\hat{f}}_{b,L}\left(x\right)=\frac{1}{bL\sqrt{2\pi }}\sum_{i=1}^{L}e^{-\frac{1}{2}{\left(\frac{x-x_{i}}{b}\right)}^{2}}.$$

The optimal bandwidth *b* was calculated following the procedure described in [26].

Although there is no consensus about the definition of the complexity, FS analysis has been employed as a statistical complexity measure [14,27].

The FS complexity C(X) is defined as the product of FIM and SEP,

C(X)=N(X)·I(X).

It can be shown that C(X)≥1, with equality if and only if *X* is a Gaussian random variable [28], which is known as the isoperimetric inequality for entropies. Moreover, it is easy to show [13] that for any scalar $a\in C^{*}$,

(7) $$\begin{array}{cc} N(aX) & ={\left|a\right|}^{2}N\left(X\right), \end{array}$$

(8) $$\begin{array}{cc} I(aX) & ={\left|a\right|}^{-2}I\left(X\right). \end{array}$$

In particular, the FS complexity of a random variable is constant under scalar multiplication.

## 4. Results and Discussion

**Figure 4:**
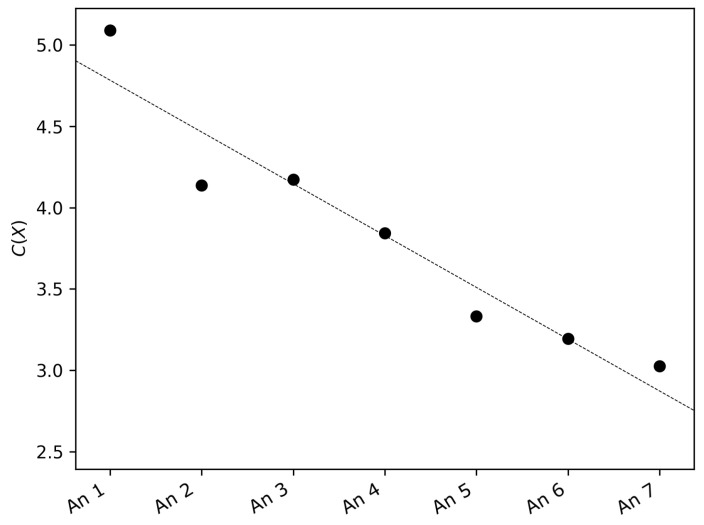
Fisher–Shannon (FS) complexity of the seven wind speed time series.

For each series, the FIM, the SEP, and the FS complexity were calculated. As an example, the bandwidth values are reported in Table 2. The FS complexity for each anemometer is shown in Figure 4. It is evident that there was a decreasing pattern of the FS complexity with height increasing from the ground. The largest value was reached at the anemometer placed at 1.5 m and the smallest for the one placed at 25.5 m. In particular, this decrease shows a non-linear relationship of wind speeds across height; otherwise, the FS complexity would be constant.

**Figure 5:**
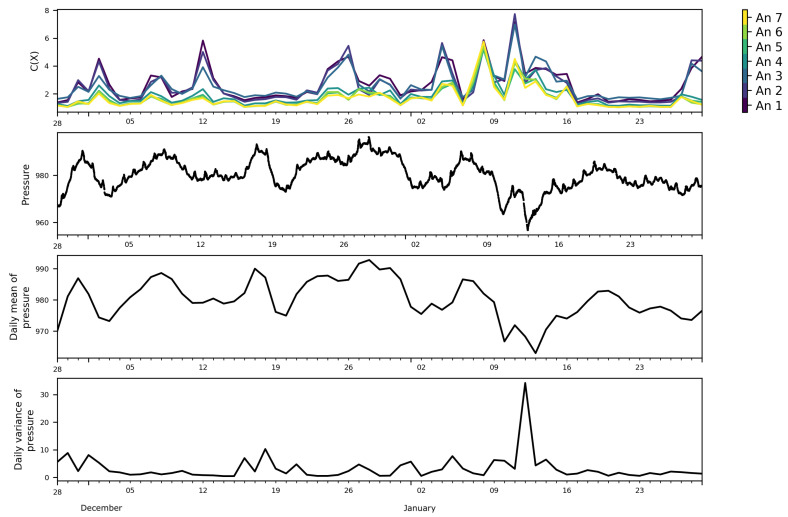
Daily FS complexity at the seven levels of the mast, pressure in (hPa), daily mean of pressure, and daily variance of pressure.

The daily variation of the FS complexity (top of Figure 5) reveals a clustering tendency among the seven anemometers: the lowest three anemometers were generally characterized by larger values of FS complexity during all of the investigation period, while the four highest ones displayed smaller values of FS complexity through time. It is interesting to note that the lowest three anemometers were placed at a height lower than the average height of the buildings surrounding the mast, while the four highest anemometers were placed well above the building average height. It is reasonable to think that such clustering of the wind series into two groups could reflect the different dynamics of the wind flow below and above the average height of the building layout [5,7,29,30]. However, all curves of the daily variation of FS complexity showed a certain coincidence of the occurrences of the peaks, especially in the last half of the investigation period.

**Figure 6:**
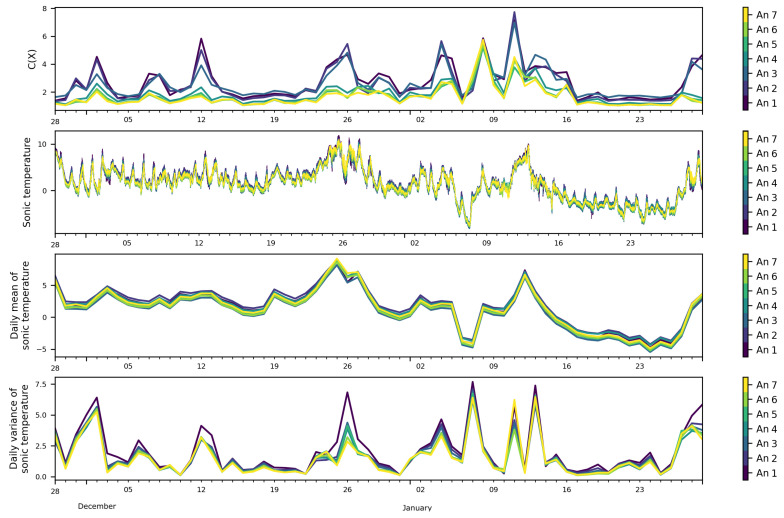
Daily FS complexity, sonic temperature in (${}^{\circ }$C), daily mean of sonic temperature, and daily variance of sonic temperature at the seven levels of the mast.

To see if any link could be found between the daily variations of the FS complexity with meteo-climatic variables, the daily mean and variance of the ambient pressure (Figure 5) and sonic temperature (Figure 6) were calculated. Showing synoptically the daily variation of the FS complexity and the daily variation of the mean pressure and sonic temperature, we can observe that most of the peaks of the FS complexity seemed to have a qualitative correspondence with those of the mean pressure, while no apparent correlation with the sonic temperature mean was observed.

**Figure 7:**
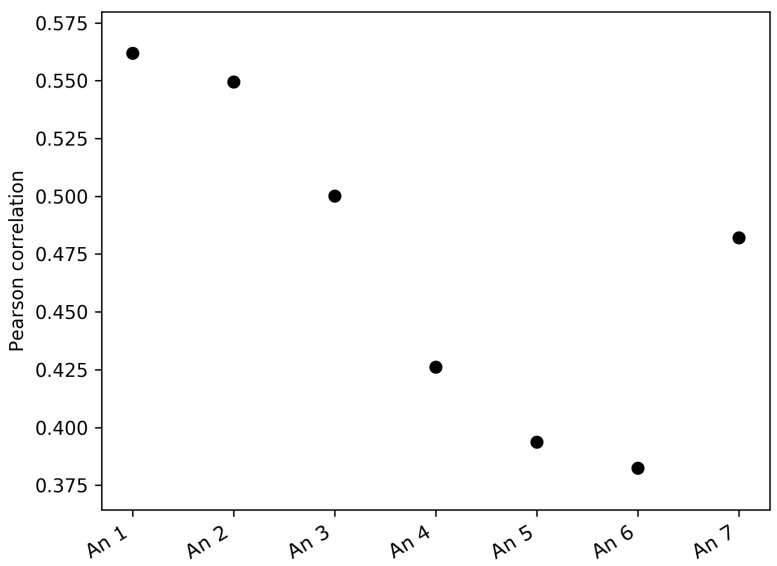
Pearson correlation between daily FS complexity and daily variance of sonic temperature at the seven levels of the mast.

The analysis of the correlations between the FS complexity and the variance of pressure and sonic temperature, instead, showed an apparent larger correlation between the FS complexity and the variance of sonic temperature, especially for the lower anemometers. Figure 7 shows that the Pearson correlation coefficients between the FS complexity and the variance of the sonic temperature were larger for the lower anemometers and smaller for the higher ones. Since the data were non-normal, a non-parametric permutation test was performed for each anemometer in order to assess the significance of the correlation coefficients [31]. The number of permutations was set to R=999. The results are presented in Table 3. It can be noted here that Anemometer 7 presented a higher correlation. However, one possible explanation could be that Anemometer 7 is in the inertial sub-layer, while Anemometers 4–6 are in the roughness sub-layer, and Anemometers 1–3 are clearly in the urban canopy layer. To verify this, in the future, longer time series will be analyzed and/or other types of vertical soundings at higher levels could help in understanding this particular phenomenon.

The analysis conducted in this study can suggest two driving forces. The atmospheric pressure obviously indicates the variation in the synoptic flows, hence giving an indication of the general weather condition during the monitoring period. While looking at the variance, the sonic temperature seemed to demonstrate that the variation of the temperature had a non-negligible effect on the complexity found in the wind speed (at least inside the canyon). The impact of the radiation and the differential heating of the surfaces inside the urban canyon could also lead to the increased variances [5]. As mentioned in Section 2, the data were analyzed for a two-month period during the winter time. In the future, this analysis could further be extended to look at the seasonal variations. However, given the driving forces, the same trends are expected.

## 5. Conclusions

An analysis of the time series of the wind speed from seven anemometers, located in an urban area on a 27-m mast at EPFL, were conducted by means of the Fisher–Shannon methods. The objective of this study was to determine the possible physical causes of the variability in the time series, but in the vertical profile of the wind. In particular, the Fisher information measure and the Shannon entropy power were applied. The study clearly demonstrated that there was a significant decrease in the complexity with height. This confirmed that the presence of buildings and more generally of obstacles in the street canyon considerably modified the wind structure and profiles in an urban setup. Additionally, the temperatures from the anemometers, as well as the atmospheric pressure from an on-site meteorological station were also used to provide data on the prevailing conditions during the monitoring campaign. It appears that the atmospheric pressure could be a proxy for the synoptic flows and the current meteorological conditions. On the other hand, the temperature is a reflection of the amount of heat brought by general flow, but also due to the heated surfaces in the canyon. This clearly points to the importance of new parameterization, particularly for the turbulent buoyancy term, as shown in [7]. Further development will include an analysis of the atmospheric stability while taking into account also the surface temperatures of the surrounding obstacles.

## Figures and Tables

**Table 1:** Summary statistics of the wind speed data in (m/s) for the seven anemometers (An).

	An 1	An 2	An 3	An 4	An 5	An 6	An 7
Height (m)	1.5	5.5	9.5	13.5	17.5	21.5	25.5
Min.	0.000	0.000	0.000	0.000	0.000	0.000	0.010
1st Qu.	0.278	0.351	0.481	0.920	1.173	1.280	1.395
Median	0.493	0.602	0.824	1.575	1.965	2.124	2.295
Mean	0.606	0.721	1.009	1.932	2.388	2.574	2.756
3rd Qu.	0.812	0.955	1.324	2.603	3.200	3.435	3.661
Max.	7.774	12.254	14.659	18.583	20.397	21.611	23.010

**Table 2:** Bandwidth values for the estimate of f(x) on the whole period.

	An 1	An 2	An 3	An 4	An 5	An 6	An 7
*b*	0.0075	0.0097	0.0138	0.0270	0.0347	0.0372	0.0404

**Table 3:** Pearson correlation coefficient and *p*-value between daily FS complexity and daily variance of sonic temperature. The *p*-values were obtained with a non-parametric permutation test (999 permutations).

	Correlation	*p*-Value
An 1	0.562	0.001
An 2	0.550	0.001
An 3	0.500	0.001
An 4	0.426	0.002
An 5	0.394	0.002
An 6	0.382	0.006
An 7	0.482	0.001

## Abbreviations

The following abbreviations are used in this manuscript:

An	Anemometer
FS	Fisher–Shannon
FIM	Fisher Information Measure
SEP	Shannon Entropy Power
pdf	probability density function
EPFL	Ecole Polytechnique Fédérale de Lausanne
